# The Mistletoe Bough

**Published:** 1871-06

**Authors:** 


### THE MISTLETOE BOUGH,

Like the Spanish moss, which drapes the trees of southern swamps in such sad funereal garb, is a growth outside of the natural condition of the tree ; it is a parasite, a fungus, a very low form of life, exceedingly slow in development in some cases, in others so inconceivably rapid as to be reproduced in millions in a few hours ; also the toadstool and mushroom. The common yeast, with which we make our bread, is a mass of living things, a dozen of them generating myriads more in a night. These fungi, sporules or germs, are not only the pests of living plants, eating out their entire life in the course of time, but they infest animals and man, carrying with them, sometimes, the most dreadful deaths. The mushroom, the morel, and the truffle, among the greatest delicacies of the table with some, are fungi. In some cases they kill, or cause disease, or poison. Ergot, blight, mildew, rust, brand, dry rot, are all the diseased results of fungous growth.

There are similar growths or products in the animal world, called "cell" life. Vegetables come from seed, animals from eggs by cell development, and these cells or eggs are as amazing in their fecundity as fungous growth. A man swallows a few mouthfuls of raw pork in which are a few

TRICHINÆ.

In a very few days living things are found burrowing in the flesh by millions, causing the most agonizing pains and a dreadful death.

Between the effects of fungi and cell products, the vegetable germ and the animal egg, men perish in millions every year.

ASIATIC CHOLERA

Seems to be, by the latest researches, the product of a thing of life, but whether of vegetable or animal admits of question thus far.

WHOOPING COUGH

Is apparently of vegetable growth; for when the expectoration of a child suffering from it is examined, it is crowded with germs; on one occasion a small amount of it was introduced into the windpipe of a healthy young rabbit; in a few days it had a troublesome cough, and on examination a countless multitude of these same germs were found all along the throat, windpipe, and lungs.

PLAGUE AND PESTILENCE,

And all those diseases which suddenly fall upon a whole community, called epidemic, such as fever and ague, chill and fever, bilious fever, yellow fever, diarrhœa, and dysentery, are caused by

MARSH MIASM.

In the worst times of yellow fever and cholera in New Orleans, the evening and the morning air was so cool and delicious and balmy that many a time we have breathed it by the hour in perfect delight; and yet the resident knew that it was but the sure intimation that the disease would be more fearful in a day or two. But if this air is bottled and taken a thousand miles away, put into a close room where a healthy man is sleeping, he will have the ordinary symptoms of chill and fever in a day or two, and myriads of these pestiferous things will be found about his tongue, his throat and windpipe and lungs and stomach.

The newspapers announced recently that the Asiatic cholera had made its appearance in India; its progress has been always westward along the most prominent lines of travel, until it reaches America, crosses to the coast of California, and is lost in the boundless Pacific.

A COURAGEOUS

Editor in the west, on the Ohio, determined to test for himself whether cholera was "catching" or not. He went on board a steamboat and found a state-room from which a man had just been taken who had died of cholera, entered, shut the door, wrapped himself up in the same bed-clothing, went to sleep, and observed no appreciable ill effects afterwards. But the dead man had been taken good care of; everything was kept scrupulously clean; the moment anything was passed from the bowels, it was thrown into the river, the vessels were rinsed well, and not an atom was allowed to fall on the bed-clothing.

If the heroic editor had slept in soiled bed-clothing, and half filled vessels had been left in the state-room all night, the present state of information points to the conclusion that the germ would have multiplied by millions during the night, and would have been drawn into the mouth and throat and stomach and lungs, to infect the system and destroy it in a few hours.

Then we come to the broad fact of special personal interest to every reader: that in case of exposure to a cholera atmosphere, and in case of having persons ill from cholera under the same roof, the one indispensable, universal necessity and safeguard, the great life preserver, is, to instantly remove out of the room, out of the house, without a second's delay longer than is necessary, every particle of what passes from the body of the patient, not allowing one drop or atom to fall on the bed-clothing; for the instant this matter is exposed to the atmosphere, as if the air contained in it some fertilizing influence or power, these germs begin to multiply rise into the air, and are taken into the body, and the increase is in geometrical progression. If the first ten minutes produces ten thousand the next will produce twenty, doubling all the time. Do not empty the vessels into the backyards, as people in the country are apt to do; nor into privies, as is done by those living in villages and towns; for the living things thrive on the air, and multiply amazingly; nor should persons in cities be content with emptying the vessels into the sinks and water-closets, and merely washing them away with water from the pipes; because cold does not kill the germ. It may be frozen solid for a year or century, but like a snake can be warmed into life. And we do not know that if cut into a thousand pieces the life would be destroyed; each piece might make a new one, and instead of killing one, that one would be multiplied by tens and hundreds.

Multitudes of things, of mixtures and preparations, have been recommended in past times as good to remove bad smells, odors, emanations, and scents; a pound of copperas dissolved in a pail of water, chloride of lime, and other things have been used to "sweeten" the air, to remove noisome smells; but all they do is to nullify what exists at the time; the spots on which they are thrown, and the piles of garbage, still go on decomposing, and more effluvia arises and new applications must be made; but as far as our present knowledge extends there never has been found any general practicable method of removing the odors and preventing more from arising in their place, until the discovery of

CARBOLIC ACID

As applicable to the needs of the case. This substance is essentially the soot which is found in chimneys carrying away the smoke from wood fires; it is creosote; it is the identical substance which was made in the old-fashioned smoke-houses with piles of little chips set on fire, and not allowed to burn, permeating the meats, and so marvelously preserving the hams and bacon, that they kept sound and good to eat for many months afterwards.

This carbolic acid is in the form of small white crystals, and is made for use by dissolving one pound of it in a hundred pounds or pints of water, to be thrown over all filthy places. It does two things, — removes the smell, and arrests decomposition indefinitely, and thus prevents any more odors from arising.

The uses to be made of it in cholera times are, to rinse every vessel with it, used to hold what passes from the body of a person suffering from cholera, and to be thrown down sinks and privies and water-closets immediately after having been used; whether it burns up the germ or egg or cell or fungi, or kills it, is yet to be determined.

Without having any experience, but reasoning from general principles, it is here suggested as a more universally available remedy, that the application of boiling water is a means of destroying the living things which are found in the passages, or as doctors say, the "dejections" of cholera patients, by pouring it on these dejections, and to make it more sure, rinsing the vessels with boiling water also, and pouring a gallon or two of boiling water down every sink, water-closet and privy the very first moment after use. So much for association of ideas in connection with

The mistletoe bough
That hung on the castle wall
When the young bride
Jumped
Into the chest in play,

"which" chest locked itself, locked her in, locked the world out, with the result that the beautiful creature was killed, suffocated and smothered to death, and dried up into a bag of bones, — poor thing!

